# Human atherosclerotic plaque transcriptomics reveals endothelial beta-2 spectrin as a potential regulator a leaky plaque microvasculature phenotype

**DOI:** 10.1007/s10456-024-09921-z

**Published:** 2024-05-23

**Authors:** Timo Rademakers, Marco Manca, Han Jin, Tanguy Orban, Ljubica Matic Perisic, Hubertus J. M. Frissen, Frank Rühle, Petra Hautvast, Jos van Rijssel, Kim van Kuijk, Barend M. E. Mees, Carine J. Peutz-Kootstra, Sylvia Heeneman, Mat J. A. P. Daemen, Gerard Pasterkamp, Monika Stoll, Marc A. M. J. van Zandvoort, Ulf Hedin, Franck Dequiedt, Jaap D. van Buul, Judith C. Sluimer, Erik A. L. Biessen

**Affiliations:** 1 Department of Pathology, Experimental Vascular Pathology Group, Maastricht University, PO box 5800, 6202 AZ Maastricht, The Netherlands; 2 Department of Plasma Proteins, Laboratory for Molecular Cell Biology, Sanquin Research and Landsteiner Laboratory, Amsterdam, The Netherlands; 3 Laboratory of Protein Signaling and Interactions, GIGA, Liège Université, Liège, Belgium; 4 Division of Vascular Surgery, Department of Molecular Medicine and Surgery, Karolinska Institutet and Karolinska Hospital, Stockholm, Sweden; 5 Genetic Epidemiology, Institute of Human Genetics, University of Münster, Münster, Germany; 6 Department of Vascular Surgery, Maastricht University Medical Center, Maastricht, The Netherlands; 7 Centre for Cardiovascular Science, University of Edinburgh, Edinburgh, UK; 8 Department of Pathology, Academic Medical Center (AMC), Amsterdam, The Netherlands; 9 Laboratory of Clinical Chemistry and Haematology, University Medical Center Utrecht, University of Utrecht, Utrecht, The Netherlands; 10 Maastricht Center for Systems Biology (MaCSBio, Cardiovascular Research Institute Maastricht (CARIM), Maastricht, The Netherlands; 11 Department of Biochemistry, Maastricht University, Maastricht, The Netherlands; 12 Department of Molecular Cell Biology, Cardiovascular Research Institute Maastricht (CARIM), Maastricht University, Maastricht, The Netherlands; 13 Department for Renal and Hypertensive, Rheumatological and Immunological Diseases (Medical Clinic II), RWTH Aachen, Aachen, Germany; 14 Institute for Molecular Cardiovascular Research, RWTH Aachen, Aachen, Germany

**Keywords:** Atherosclerosis, Plaque microvessels, Leaky vessels, Stiffness, Vascular biology

## Abstract

The presence of atherosclerotic plaque vessels is a critical factor in plaque destabilization. This may be attributable to the leaky phenotype of these microvessels, although direct proof for this notion is lacking. In this study, we investigated molecular and cellular patterns of stable and hemorrhaged human plaque to identify novel drivers of intraplaque vessel dysfunction. From transcriptome data of a human atherosclerotic lesion cohort, we reconstructed a co-expression network, identifying a gene module strongly and selectively correlated with both plaque microvascular density and inflammation. Spectrin Beta Non-Erythrocytic 1 (*sptbn1*) was identified as one of the central hubs of this module (along with *zeb1* and *dock1*) and was selected for further study based on its predominant endothelial expression. Silencing of *sptbn1* enhanced leukocyte transmigration and vascular permeability in vitro, characterized by an increased number of focal adhesions and reduced junctional VE-cadherin. In vivo, *sptbn1* knockdown in zebrafish impaired the development of the caudal vein plexus. Mechanistically, increased substrate stiffness was associated with *sptbn1* downregulation in endothelial cells in vitro and in human vessels. Plaque SPTBN1 mRNA and protein expression were found to correlate with an enhanced presence of intraplaque hemorrhage and future cardiovascular disease (CVD) events during follow-up. In conclusion, we identify SPTBN1 as a central hub gene in a gene program correlating with plaque vascularisation. SPTBN1 was regulated by substrate stiffness in vitro while silencing blocked vascular development in vivo, and compromised barrier function in vitro. Together, SPTBN1 is identified as a new potential regulator of the leaky phenotype of atherosclerotic plaque microvessels.

**Supplementary Information:**

The online version contains supplementary material available at 10.1007/s10456-024-09921-z.

## Introduction

In the last decades, our understanding of atherosclerosis has vastly increased, and a myriad of novel insights has been gained on the various players that come together in atherosclerosis, i.e. lipid transportation/metabolism, inflammation, and plaque neovascularization. Atherosclerotic plaque angiogenesis is correlated with increased plaque progression, and has been recognized as a double edged sword in the development of advanced, instable atherosclerotic lesions [1]. On the one end, afflux of oxygen to the lesion could alleviate plaque hypoxia, leading to a reduction in e.g. chronic oxidative stress [2]. Yet, in practice, these plaque vessels have also been associated by us and others with increased permeability, leakage of erythrocytes and lipids, and recruitment of inflammatory cells, both in human and murine lesions [3–6]. We have previously shown that murine plaque-associated microvessels (vasa vasorum) are more permeable than similarly sized control microvessels, displaying more pronounced leukocyte adhesion to the local vessel wall, with a concomitant increase in leukocyte transmigration [7]. The molecular changes that underlie this dysfunction in atherosclerosis are however only poorly understood, especially in human disease, which is not always (completely) reflected by murine studies.

Several factors have long been associated with a leaky phenotype, amongst which growth factors like VEGF [8]. Especially VEGF is well known not only for its role in the angiogenic process, but also in vascular hyperpermeability. In addition, the Tie2/Angiopoietin pathway is well-studied in relation to vessel maturation [9], and also several other growth factors or receptors, e.g. NRP-1 [10] have been implicated in a leaky vessel phenotype. The underlying common mechanisms are local changes in the endothelial cells themselves, promoting permeability and increased leukocyte adhesion.

Recent studies, most notably from the oncology field, have proposed stabilization of unstable, leaky vessels as therapeutic strategy to slow down or prevent disease progression. This may be achieved either by reinstating their barrier function through normalization of their maturation or as recently suggested, by limiting endothelial metabolism [11]. This is an attractive solution to avoid straightforward inhibition of pathological plaque angiogenesis, which is undesirable as evident from the above-described functional ambiguity. Moreover, targeting well-known growth factors, e.g. anti-VEGF treatment, also interferes with the growth factors’ systemic roles, causing variable results in therapeutic interventions [8, 12]. Ideally, therapeutic targets should be more disease-specific, and linked to the local pathological response. It descends from the above, the necessity to identify specific players that are altered in human plaque microvessels.

In this study, we set out to identify potential proteins that are specifically regulated in plaque microvessels in human atherosclerotic lesions. To get a better overview of how the pathological angiogenic response in atherosclerosis is governed, we applied network coexpression analysis of transcriptomic data obtained from well-characterized human atheromas, to pinpoint critical factors in increased angiogenesis and/or the leaky phenotype of plaque microvessels and validate their role in the regulation of microvessel patency in the context of atherosclerosis.

## Methods

### Patient samples, histology, and immunohistochemistry

Paired stable and unstable segments from human atherosclerotic plaque samples were obtained from carotid artery lesions from 24 patients undergoing endarterectomy (Department of Vascular Surgery, Maastricht University Medical Center, Maastricht, the Netherlands) as part of the Maastricht Pathology Tissue Collection (MPTC). Collection, storage, and use of tissue and patient data were performed in agreement with the Dutch Code for Proper Secondary Use of Human Tissue. Plaque segments were staged by histological analysis based on HE staining according to Virmani et al., where pathological intimal thickening (PIT) was classified as early lesions, thick fibrous cap atheroma (TkFCA) were classified as advanced stable, and intraplaque hemorrhage (IPH) as ruptured (advanced unstable) segments, respectively [13].

Immunohistochemical stainings were performed on consecutive paraffin sections for CD31 (vascular endothelial cells), CD105 (angiogenic marker), αSMA (smooth muscle cell/pericyte), the CD68 (macrophages), and SPTBN1. Appropriate IgG control antibodies were used as a negative control. Double stainings were performed for CD31/CD105, αSMA/CD31, and CD31/SPTBN1 and analyzed using spectral imaging system.

### Cell culture

Human umbilical vein endothelial cells (HUVEC) or the human microvascular endothelial cell line (HMEC-1), derived from human foreskin endothelial cells, were cultured on fibronectin (FN) coated plates in respectively EGM2 or RPMI1640 + glutamax. HUVECs were used for experiments between P2 and P4, HMEC-1 cells were used until a maximum of P20. For knockdown of specific genes, cells were treated for 4 h with a targeted siRNA in combination with HiPerfect transfection reagent in Opti-Mem, and cells were used for various assays after 24–48 h post-transfection.

### RNA extraction & transcriptomics on patient samples

RNA isolation was performed by Guanidium Thiocyanate lysis followed by Cesium Chloride gradient centrifugation and purified using the Nucleospin RNAII kit. RNA concentrations were measured, and RNA quality and integrity was determined using Lab-on-Chip analysis. Biotinylated cRNA was prepared from 100 ng total RNA using the Illumina TotalPrep RNA Amplification Kit according to the manufacturer’s specifications. 750 ng of cRNA per sample was hybridized to Illumina Human Sentrix-8 V2.0 BeadChip® and washed according to the Illumina standard procedure. Scanning was performed on the Illumina BeadStation 500, image analysis and extraction of raw expression data was performed with default settings and without normalization.

### Computational methods

Analyses of transcriptomic data were performed in R [14]. Raw expression data were imported using the package *lumi* [15], and normalized by robust spline normalization. Co-expression networks were estimated by applying the methods implemented in the package *WGCNA* [16]. Module-traits association was estimated by Pearson correlation of the expression modules eigenvalues and quantitative traits. Network architecture was visualized using Cytoscape [17] and Db-String. ClueGO was used to enrich the modules of interest for overrepresented Gene Ontology terms and Pathways [18].

In order to reveal the relationships between proteins/peptides and microvessel density (MVD), a ranking list was performed to show the importance of proteins/peptides relating to MVD based on two different measurements, the Pearson Correlation Coefficient and the Maximal Information Coefficient (MIC) [19]. In each ranking list, top-100 high-ranked proteins/peptides were listed, after which overlapping proteins/peptides were marked and cross-referenced with hemorrhage-related proteins.

### Zebrafish

*Tg(fli1a:eGFP)y1* embryos were injected with control morpholino (Ctrl Mo) or with morpholino against SPTBN1 (7.5ng/microliter) at the one-cell stage (n = 35 per group). Confocal pictures of the caudal vasculature were taken at 48 h post-fertilization (hpf) using a Zeiss confocal microscope. Caudal vein plexus (CVP) thickness and area were analyzed using ImageJ software.

### Statistical analysis

Where not explicitly specified otherwise, all data are presented as mean ± SEM. For patient data, groups were compared using a Mann–Whitney rank-sum test for continuous variables. For correlation analyses of human qPCR and protein expression data, Shapiro–Wilk test for normality was performed, after which correlations of Gaussian distributed data were calculated by Pearson and of non-Gaussian data by Spearman correlation test. For group comparisons, data were tested for Gaussian distribution, after which a Student’s t-test (Gaussian) or Mann–Whitney U test (non-Gaussian) was used to compare individual groups; multiple groups were compared by ANOVA or Kruskall-Wallis tests, with Bonferroni or Dunn’s post-hoc test, respectively. Statistics were performed using Graphpad Prism 5.0. A p-value of < 0.05 was considered statistically significant. *, **, and *** denote p < 0.05, p < 0.01, and p < 0.001, RESP.

Full materials and methods are available in the online supplement.

## Results

### Microvessel density was increased in unstable advanced plaque segments and associated with increased CD105, whereas perivascular coverage was unaltered

**Fig. 1:**
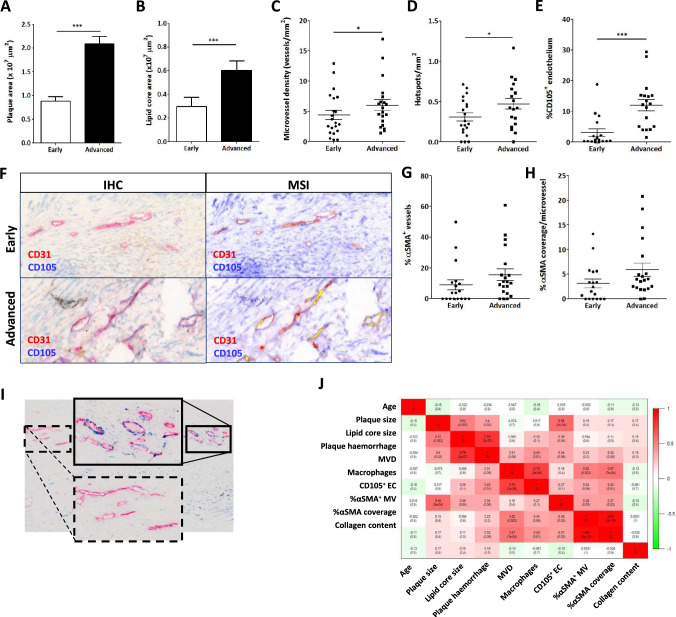
Intraplaque microvessel characteristics differ between advanced stable and advanced unstable atherosclerotic lesions. **A** + **B** Plaque (**A**) and lipid core (**B**) size of the Maastricht Human Plaque Study cohort were determined and were, as expected, significantly increased in advanced lesions with hemorrhage compared to the stable early lesions. **C** + **D** Concomitantly, there was an increase in both microvessel density (**C**) and the number of microvascular hotspots (**D**) within the advanced lesions. (**E** + **F**) The percentage of angiogenic endothelial cells (CD105 + CD31^+^) was determined by double staining for CD105 and CD31 and subsequent multispectral imaging (MSI) and unmixing of the CD105 and CD31 signals. Image analysis showed that the proportion of CD31 + endothelial cells that were also positive for CD105 + (angiogenic EC) was significantly increased in advanced versus early plaques (**E**). Representative images of the double staining are presented in panel F. **G** + **H** Stability of the microvessels, assessed by percentage of vessels covered by αSMA^+^ smooth muscle cells (SMC, **G**), as well as the amount of SMC coverage per vessel (percentage αSMA-positivity, H) were not significantly different. **I** This lack of effect may be, in part, caused by the high variability in the degree of αSMA-coverage between different CD31^+^ hotspots. **J** We did not observe significant correlations between MVD and angiogenesis (CD105^+^) or vessel maturation αSMA^+^. Data are from 22 patients (both stable and unstable advanced lesions) (**A**–**J**); error bars indicate SEM (**A**–**E**, **G**, **H**); *p ≤ 0.05; **p ≤ 0.01; ***p ≤ 0.005. Scalebars indicate 20µm

To assess plaque and lipid core size, paraffin-embedded, hematoxylin–eosin stained sections were quantified morphometrically, revealing an increased plaque (Fig. 1A) and lipid core size (Fig. 1B) in advanced unstable (with intraplaque hemorrhage) segments compared to segments with an early, stable phenotype of the same symptomatic patient. Staining for the vascular marker CD31 showed both increased microvessel density (MVD) (Fig. 1C) and microvessel hotspots (Fig. 1D) within the plaque. Co-localization of the angiogenic marker CD105 and the vascular marker CD31 showed an augmented percentage of angiogenic plaque microvessels in advanced unstable lesions (Fig. 1E), as assessed in using multispectral analysis (Fig. 1F). Perivascular coverage defined as αSMA-covered microvessels (Fig. 1G) and the amount of perivascular coverage (αSMA-positivity) per microvessel (Fig. 1H) were measured but did not show differences between early and advanced plaque segments. This may in part be due to the high degree of heterogeneity in perivascular coverage observed within lesion segments (Fig. 1I), and/or symptomatic nature of the patient. Analysis of the cross-correlation between plaque traits suggested a strong association of MVD with macrophages and αSMA-coated mature vessel presence, but not with CD105^+^ angiogenic endothelial cells (EC). (Fig. 1J). Plaque content of CD105^+^ angiogenic ECs was seen to correlate with plaque size and, at borderline significance, with lipid core size and intraplaque hemorrhage.

### Analysis of the gene cluster highly correlating with MVD, but not with angiogenic activity, yielded potential target genes involved in plaque neovascularization

**Fig. 2:**
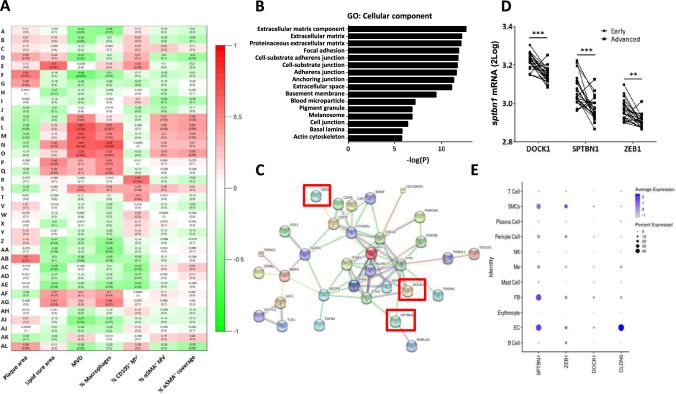
Microarray analysis shows clear correlation of MVD with specific modules, which are enriched for GO terms related to endothelial junctions and cytoskeletal organization. **A** Heatmap of the module-trait correlations, showing upregulated (red) or downregulated (green) gene modules in relation to phenotypic traits in advanced unstable lesions. A high overlap in genetic profile was found for microvessel density (MVD) and lesional macrophage content (% Macrophages), while the percentage angiogenic endothelium (% CD105^+^ MV) showed a different expression profile. αSMA parameters showed certain overlap with MVD but failed to show highly enriched modules. Values of the Pearson's r coefficient and associated p-values (in parenthesis) are reported. **B** GO analysis on the overrepresented module shows a clear enrichment of biological processes related to endothelial cell adhesion, endothelial junctions, and extracellular matrix and cytoskeletal organization. **C** PPI network reconstruction for the most central and interconnected gene members of the MVD correlated module L revealed a network which included several known endothelial key proteins. Highlighted are the three hub genes showing the highest ranks based on ranking analysis, which were selected for further study: *zeb1, sptbn1* and *dock1* (enboxed). **D** Interrogation of single cell RNASEq dataset of Alsaigh et al. [20] revealed preferential expression of *sptbn1* and to a lesser extent *zeb1* and *dock1* by plaque endothelial cells. Average expression is color coded (white to blue), node size reflects % of cells with target gene expression. The profile of the endothelial signature gene *cldn5* profile is added to serve as reference

To gain mechanistic insights into the process that link microvessel density to plaque destabilization, we analyzed the microarray dataset from our study population by weighted gene coexpression network analysis (WGCNA). This revealed 38 gene modules overall. The module’s Eigengenes were subsequently correlated to the histological traits e.g. MVD, angiogenic activity, and perivascular coverage of plaque microvessels, after which correlations were visualized in a module-trait heatmap (Fig. 2A). MVD was highly correlated with module L (P = 1.0 × 10^–4^), and angiogenic activity (CD105^+^) with module R (P = 0.004). As shown earlier, MVD and lesion macrophage content showed considerable overlap in module correlation pattern, in support of inflammatory regulation and/or influx from leaky microvessels. Hence, we opted for module L. Gene ontology (GO) analysis of this module revealed a clear enrichment of biological processes related to cell- and matrix-adhesion, and wound healing (Fig. 2B), next to overrepresentation of extracellular matrix, focal adhesion, and junctional components (GO: cellular components). To select candidate genes for further study, we next ranked the genes within module L by highest correlation for MVD and highest centrality within the module’s subnetwork (Supplemental Table 1). A protein–protein interaction network constructed for the top-ranking (hub) genes using STRING (Fig. 2C) confirmed the overrepresentation of factors related to endothelial cell function, including VE-cadherin, vinculin, and paxillin. Interrogation of the human plaque single cell RNASeq datasets, showed that SPTBN1 and to a lesser extent also DOCK1 and ZEB1 were mainly expressed by endothelial cell subsets (dataset Alsaigh et al. [20], Fig. 2E; metadata Mosquera et al. [21], Supplemental Fig. 1A). Based on their association with the protein–protein interaction network and the relative ranking, in combination with the single cell expression, we selected Dedicator-Of-Cytokinesis 1 (*dock1*), Spectrin Beta Non-Erythrocytic 1 (*sptbn1*), and Zinc Finger E-Box Binding Homeobox 1 (*zeb1*), for further study of their function in endothelial cells.

### Knockdown of *sptbn1* and *zeb1* both impact endothelial function

**Fig. 3:**
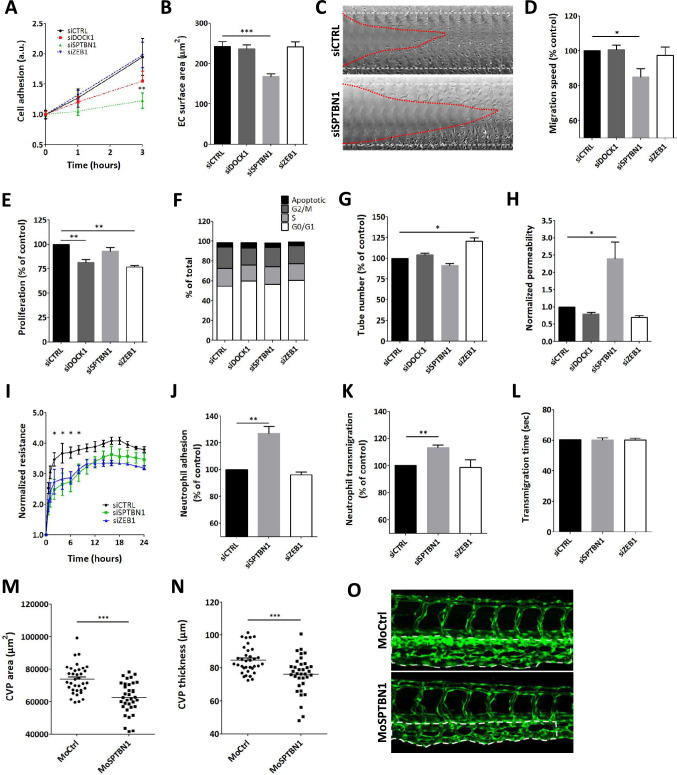
Knockdown of *dock1*, *sptbn1*, and *zeb1* reveal a range of functional effects. **A** Endothelial cell adhesion was significantly altered for *sptbn1* knockdown, whereas *dock1* knockdown showed an intermediate phenotype and siZEB1 was ineffective. **B** For *sptbn1*, the reduced cell adhesion is accompanied by defective cell spreading. **C** + **D** In addition, *sptbn1* knockdown also reduced endothelial migration speed in a wound healing assay (**C**: example; **D** quantitative data). **E** Endothelial proliferation rate was impaired after *dock1* and *zeb1*, but not *sptbn1* knockdown; **F** cycle stage analysis did not reveal significant shifts in cell cycle stage. **G** Tube formation was slightly increased upon *zeb1* knockdown, with a trend towards reduced tube formation in *sptbn1* knockdown cells. **H** As a functional parameter, we assessed permeability for 150kD dextran in a Transwell system, revealing a significant increase in permeability upon knockdown of *sptbn1*, whereas *dock1* or *zeb1* silencing was ineffective. **I** In keeping, we observed decreased resistance of the endothelial monolayer upon *sptbn1*1 but not *zeb1* knockdown. **J**–**L** Furthermore, *sptbn1* but not *zeb1* knockdown also increased adhesion (**J**) and transmigration (**K**) of neutrophils under flow, while overall transmigration time (**L**) was unaffected. Based on these findings SNTBN1 was taken for in vivo validation in zebrafish. **M**, **O** Microinjection of zebrafish embryo’s with morpholino antisense oligonucleotides against *sptbn1* led to a significant reduction in caudal vein plexus (CVP) development in zebrafish, as shown by the CVP area (M) and CVP thickness (**N**). Representative images of control and *sptbn1* in zebrafish show de reduced CVP area and thickness (**O**, dotted area). Data are from three (**A**–**G**, **I**–**L**) or four (**H**) independent experiments or 35 zebrafish per group (**M**–**O**); error bars indicate SEM (A-C, E-N); *p ≤ 0.05; **p ≤ 0.01; ***p ≤ 0.005

First, we confirmed solid and preferential expression of the candidate genes in HMEC-1 *(*Supplemental Fig. 1B*)* endothelial cells in vitro compared to (polarized) THP-1 cells and primary vascular smooth muscle cells. We confirmed effective siRNA silencing of the target genes at mRNA and protein level *(Supplemental *Fig. 1C-D*)*. Subsequently, loss-of-function studies in HMEC1 were performed to assess the impact of the candidates on critical endothelial functions, including EC adhesion, spreading, migration, proliferation and cell cycle analysis, tube formation capacity, vascular permeability, and leukocyte transmigration under flow (Fig. 3). *Dock1* knockdown did not result in major differences throughout these assays (Fig. 3A-H), with only cell proliferation being significantly affected (Fig. 3E, F). *Zeb1* knockdown also significantly reduced proliferation (Fig. 3E, F), and in addition affected tube formation capacity, with increased tube number (Fig. 3G). Knockdown of *sptbn1* however showed a profound reduction of cell adhesion (Fig. 3A), spreading (Fig. 3B), and migration (Fig. 3C, 3D). Moreover, it dramatically altered vascular permeability in a dextran leakage Transwell assay (Fig. 3H), a finding that could be confirmed in the transendothelial electrical resistance (TEER) assay (Fig. 3I). Considering the marginal functional effects of *dock1* silencing, it was excluded from subsequent analysis. Silencing of s*ptbn1* but not *zeb1* augmented leukocyte adhesion to the endothelium under flow (Fig. 3J), as well as leukocyte transmigration (Fig. 3K), although the rate of leukocyte transmigration was unaltered (Fig. 3L). This led us to focus on *sptbn1* as prime candidate for in vivo validation of its regulatory role in vessel function in vivo. In vivo knockdown of *sptbn1* by microinjection of morpholino oligonucleotides into zebrafish embryos led to attenuated development of the caudal vein plexus (CVP) (Figs. 3M-O), in line with the reduced adhesion, spreading, and migration in vitro.

### SPTBN1 expression is regulated by tissue stiffness in vitro and in vivo

**Fig. 4:**
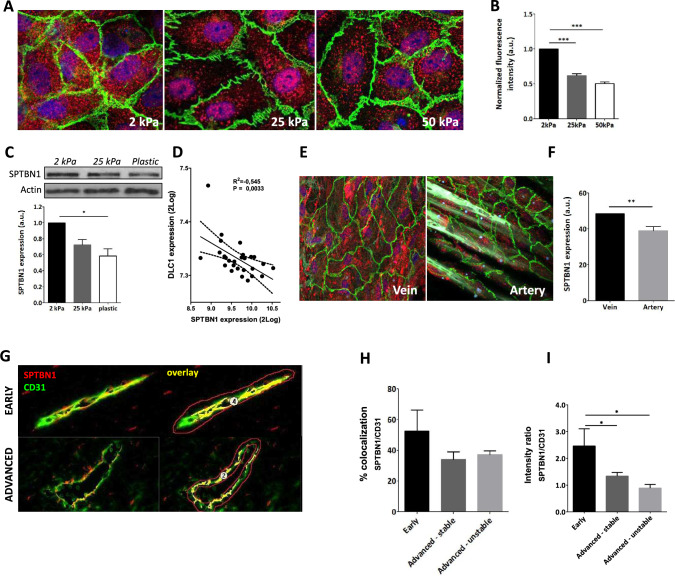
Increased stiffness reduced SPTBN1 protein expression both in vitro and in vivo. **A** HUVEC cultured on different stiffness gels showed reduced SPTBN1 protein expression by immunofluorescent staining (red). **B** Quantification of the IF staining showed a clear decrease in expression of SPTBN1 protein with increasing stiffness. **C** A similar stiffness associated reduction in SPTBN1 protein expression by endothelial cells was observed after Western Blot. **D** To confirm that SPTBN1 levels are stiffness dependent, the SPTBN1 expression was correlated to the stiffness sensitive marker DLC1, clearly showing reduced SPTBN1 expression upon higher stiffness. **E** + **F** To determine whether reduced SPTBN1 expression upon lower stiffness is relevant in vivo, we assessed mesenteric vein and artery from the same patient for SPTBN1 protein expression by immunofluorescence staining; a representative image is presented in **E**. We found that the softer vein had significantly higher endothelial expression of SPTBN1 compared to the stiffer artery (**F**). **G**–**I** Multispectral imaging of endothelial cells double stained for CD31 (green) and SPTBN1 (red) (G for representative images, costaining is given in yellow). Image analysis revealed a slight but not significant reduction in the level of colocalization of CD31 and SPTBN1 in microvessels in advanced vs early lesions (**H**), whereas relative SPTBN1 expression in plaque microvessels was significantly and progressively reduced with disease progression (**I**). Data are from three (**A**–**D**) or four (**E**, **F**) independent experiments or from 20 patient samples (**G**–**I**); error bars indicate SEM (**B**, **C**, **D**, **F**, **H**, **I**); *p ≤ 0.05; **p ≤ 0.01; ***p ≤ 0.005. Scalebars indicate 10µm (**A** and **E**), or 20µm (**G**)

While expression of *sptbn1* was reduced in advanced atherosclerosis, and linked to microvascular permeability in vitro, its regulation in the context of atherosclerosis is unclear. Relevant stimuli, including LPS induced inflammation, oxLDL exposure, or a combination of both, did not significantly alter *sptbn1* mRNA expression in HMEC-1. As our network and the network predictions of module L (Fig. 2C) revealed substrate stiffness-responsive genes, we studied the role of this stimulus on *sptbn1* expression. As HMEC-1 did not grow well on the stiffness gels, we assessed in HUVECs what effect substrate stiffness would have on *sptbn1* expression. Total SPTBN1 protein expression was progressively decreased with increasing substrate stiffness, as judged by immune fluorescence staining (Fig. 4A, red; Fig. 4B), as well as by Western Blotting analysis (Fig. 4C). Furthermore, we could observe a clear inverse correlation between SPTBN1 and the stiffness-sensitive marker DLC1 [22] (Fig. 4D), and in primary tissue, softer venous tissue showed a higher SPTBN1 expression compared to stiffer arterial tissue (Fig. 4E, 4F). Next, we performed multispectral imaging, examining colocalization of SPTBN1 with the vascular marker CD31 in plaque microvessels (Fig. 4G), to dissect the expression of endothelial SPTBN1 during disease progression. Interestingly, the level of colocalization between SPTBN1 and CD31 decreased with disease progression (Fig. 4H). In addition, the local expression levels of SPTBN1 in plaque vessels in more advanced stable and unstable lesions were significantly reduced compared to early lesions (Fig. 4I). As plaque presence lowers the elasticity of the vessel wall and increased local stiffness, especially at later stages, the reduced expression of SPTBN1 in plaque microvessels may be attributable to the biomechanical properties of plaque tissue.

### SPTBN1 is involved in gene networks related to cell–cell junctions and cell–matrix interactions

**Fig. 5:**
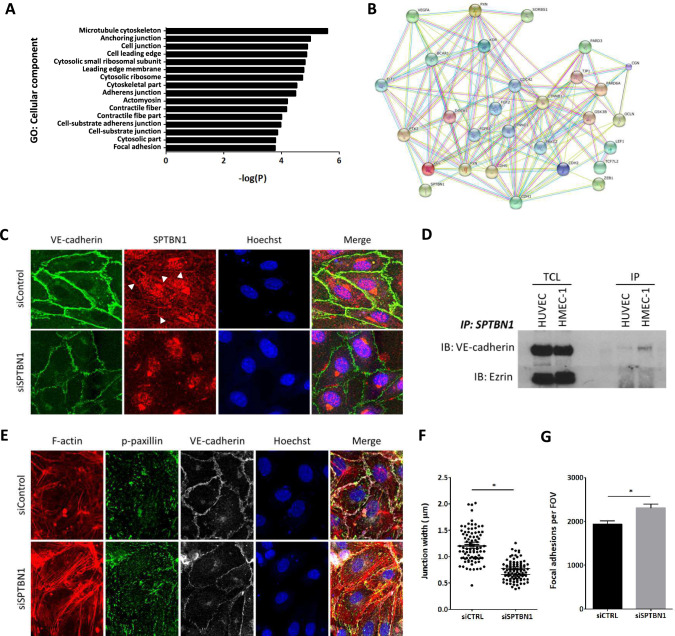
SPTBN1 has a potential role in cell junction regulation, is associated with VE-cadherin at endothelial cell junctions and is linked to focal adhesion regulation. **A** *Sptbn1* was silenced in HMEC-1, after which gene expression was measured by microarray analysis. GO analysis of the differentially expressed genes revealed links to regulation of adherens junctions and cell junctions, as well as focal adhesions. **B** Subsequent network prediction analysis showed clear involvement of *sptbn1*, but also confirmed its connection with *dock1* and zeb1 in these pathways, and its link to junctional proteins like VE-cadherin (CDH5), focal adhesion proteins like paxillin (PXN), and cell–matrix related proteins like vinculin (VCL). **C** Immunofluorescent staining of HMEC1 with silenced sptbn1 (or siControl treated controls) for SPTBN1 (red) and the endothelial junction protein VE-Cadherin (green); nuclei were counterstained with Hoechst 33,342. As expected, silencing led to sharply reduced SPTBN1 expression, while interestingly also VE-Cadherin expression was reduced. The latter was seen to be more dispersed over the whole cell after sptbn1 silencing, but is enriched at stable, straight junctions between cells, as well as at the basolateral side. At junctions, SPTBN1 was seen to colocalize with VE-cadherin. **D** Immuno-precipitation analysis of HUVEC and HMEC-1 for SPTBN1, followed by immunoblotting for VE-cadherin and Ezrin (loading control) demonstrated direct interaction of SPTBN1 and VE-Cadherin for both cell types. TLC: Total cell lysate, IP is Immuno-precipitate (**E**) Knockdown of SPTBN1 led to an increase in stress fibers and an increased staining of p-paxillin at the basolateral side of the cell, indicative of an increase in the number of focal adhesions, and in all, an altered cell adhesion to the substrate (**F** + **G**) Quantitative image analysis revealed reduced junctional width after s*ptbn*1 silencing (**F**), with a concomitant increase in paxillin and focal adhesions (p-paxillin) at the basolateral side of the cell (**G**). Data are representative images from three independent experiments (**C**–**G**); error bars indicate SEM (**F**, **G**); * p ≤ 0.05. Scalebars indicate 10µm. **D**, **F**, **H**, **I**); *p ≤ 0.05; **p ≤ 0.01; ***p ≤ 0.005

The effects of *sptbn1* silencing on permeability and leukocyte transmigration, and its interaction with cell junction and adhesion proteins, point to a role of SPTBN1 in the regulation of EC function. To delineate the mode of action of SPTBN1’s regulatory function, we profiled mRNA expression patterns upon *sptbn1* silencing versus control siRNA treated and untreated HMEC-1 and HUVEC. This analysis yielded 514 differentially expressed genes, of which 47 were also contained in the MVD associated module L geneset of the human plaque study cohort. (P = 0.03; Supplemental Fig. 1E). GO analysis and protein–protein interaction network analysis of the top 40 most differential genes after *sptbn1* silencing revealed a clear overrepresentation of pathways involved in cell–cell and cell–matrix adhesion, as well as cell–cell junction and focal adhesion regulation e.g. cadherin-1, -2, and -5 (VE-cadherin), paxillin, vinculin, tight junction protein-1, and occludin (Fig. 5A & B), which was confirmed of the BIKE cohort (Supplemental Table 2*)*.

### SPTBN1 associates with VE-cadherin at the cell junction and is also involved in focal adhesion regulation

To assess the role of SPTBN1 in endothelial cells, we studied the localization of this protein. Staining of SPTBN1 on endothelial cells revealed clear presence of the protein on stable, linear junctions (Fig. 5C). Also, SPTBN1 was highly prevalent in/near the Golgi, and predominantly localized in the basolateral plane of the cell. To further investigate the association of SPTBN1 with EC junctions, we performed an immunoprecipitation (IP) of SPTBN1, and stained for junctional proteins like VE-cadherin and the tight junction protein ZO-1. VE-cadherin was clearly visible after SPTBN1 pulldown (Fig. 5D), both in HUVEC and HMEC-1, while ZO-1 was not present. These data were confirmed by IP for VE-cadherin and subsequent immunoblotting for SPTBN1 (Supplemental Fig. 2A).

Upon knockdown of *sptbn1*, we observed less linear junctions, a concomitant increase in reticular and focal adherens junctions (Fig. 5E). This was accompanied by an increased frequency of gaps between endothelial cells (Supplemental Fig. 2B), by increased stress fiber expression (Fig. 5E + F) and basolateral focal adhesions, and by reduced junctional width (Fig. 5G). Of note, we also observed a trend for higher levels of the focal adhesion protein paxillin. These data underpin the causal role of *sptbn1* knockdown in junctional instability and altered focal adhesion dynamics, potentially compromising permeability and promoting leukocyte transmigration.

### Reduced SPTBN1 expression is associated with increased intraplaque hemorrhage and hemorrhagic event risk

**Fig. 6:**
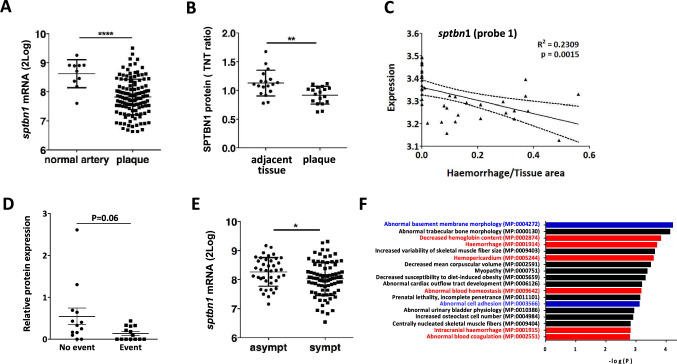
Validation of the association of SPTBN1 expression in human plaque and clinically relevant endpoints. **A** + **B** Validation of SPTBN1 expression in carotid artery plaque at mRNA (**A**) and protein level in the BIKE cohort (**B**), showing significant downregulation of SPTBN1 in plaque tissue compared to normal arteries or tissue adjacent to the plaque, respectively. To investigate the role of SPTBN1 in inflammation and vascular permeability in vivo, we assessed whether SPTBN1 expression is linked to intraplaque bleeding. **C** In the Maastricht Human Plaque Study cohort, *sptbn1* expression showed an inverse correlation with the extent of intraplaque hemorrhage, here shown for one splicing variant (probe-1) (P = 0.0015; r^2^ = 0.2309). **D** In the independent Atheroexpress cohort, plaques of patients suffering from a cardiovascular event during follow-up had fourfold lower expression of SPTBN1 protein than event-free patients (**E**). In the same BIKE cohort, SPTBN1 mRNA expression was significantly lower in plaque of symptomatic vs asymptomatic patients. **F** The peptidomics dataset of the Maastricht Human Plaque Study was analyzed for peptides with significant correlation with module L; the corresponding protein list was strongly overrepresented in proteins of associated with hemorrhage (red) as well as proteins linked to ECM and endothelial cell adhesion (blue). Data are from n = 127 (A, E; BIKE), 36 (B; BIKE), 43 (C, F, Maastricht Human Plaque Study), and 28 (**D**; AtheroExpress) patient samples; error bars indicate SD; *p ≤ 0.05, **p ≤ 0.01, ***p ≤ 0.005, ****p ≤ 0.001

Next, we assessed whether SPTBN1 downregulation contributes to leaky vessel phenotype in human disease. Hereto, we evaluated the correlation of SPTBN1 expression in human plaque with histological features such as intraplaque hemorrhage in our Maastricht Human Plaque Study (MaasHPS) cohort. S*ptbn1* expression was not only downregulated in advanced versus early lesions in our MaasHPS cohort (Fig. 2D), but also in BIKE, at mRNA (plaque versus normal artery) (Fig. 6A) and protein level (carotid artery plaque vs adjacent adjacent tissue) (Fig. 6B). Moreover, *sptbn1* mRNA expression showed a very significant inverse correlation with plaque hemorrhage size not only for the main isoform (Fig. 6C) but also for the other 2 detectable splicing variants (Supplementary Fig. 2C). This association and its clinical implications could be validated in the Athero-Express Biobank cohort, with significantly lower plaque SPTBN1 protein expression in CVD event-free patient than in patients with an CVD even during follow-up, albeit that this effect was borderline significant (P = 0.06) (Fig. 6D). Validation in the BIKE cohort showed a highly significant 40% lower *sptbn1* mRNA expression in symptomatic than asymptomatic patients (Fig. 6E). To confirm the link to hemorrhage and leakage is, we interrogated the peptidomics dataset of the MaasHPS cohort. Interestingly, almost 50% of the top 100 peptides with highest correlation with microvessel density were representing plasma proteins (P = 1.3 × 10^–9^), but the top 100 peptides were also enriched in focal adhesion and junctional cadherin binding peptides (Supplemental Fig. 2D), again confirming our earlier findings. Mammalian Phenotype Ontology analysis revealed a clear association with hemorrhage (Fig. 6F, red), while GO pathway analysis indicated an overrepresentation of platelet activation and degranulation pathways among the CD31^+^ plaque vessel correlated peptides (data not shown), consistent with hemorrhage. These observations all support a role for SPTBN1 in microvascular permeability and vulnerability in human plaques.

## Discussion

The link between human plaque angiogenesis and enhanced atherosclerotic burden and intraplaque hemorrhage has been well established. However, beyond the prototypic angiogenic factors that have been described in the field of angiogenesis, e.g. VEGF and its receptors, we still lack deeper understanding of how microvessel formation and function in—in particular—human atherosclerotic plaque are regulated. In vitro models and in vivo mouse models have limited value as the regulation of critical genes or proteins that influence vessel function may (in part) depend on the local environment in human plaque, which may not be modelled in murine models. Therefore, we used a genomics-based approach to identify new, unknown regulators of human plaque microvessel function. This novel approach in human atherosclerotic tissue allowed unbiased identification of central mediators of plaque microvascular function. Here we report SPTBN1 as a novel actor in microvessel permeability and leukocyte transmigration in vitro.

SPTBN1, or Spectrin Beta Non-Erythrocytic 1, has previously been implicated in actin crosslinking, and has been described as a scaffold/adaptor protein in actin cytoskeleton anchoring into the membrane [23, 24]. In parallel to similar scaffold proteins, SPTBN1 is therefore thought to be involved in regulating e.g. cell shape, motility, as well as compartmentalization of transmembrane proteins. SPTBN1 functions has been studied in considerable detail in brain and heart physiology. In brain, SPTBN1 found predominantly in Purkinje-cell bodies, and appear to be involved in the assembly of specialized membrane domains in Purkinje neurons [24]. In heart, SPTBN1 has been shown to be a vital part of the membrane-associated cytoskeleton of cardiomyocytes, and was shown to be pivotal for the organization of several key components in cardiomyocyte functioning [25]. More recently, Smith and al showed that lack of cardiac SPTBN1 caused altered localization of the sarcoplasmic reticulum ryanodine receptor 2 (RyR_2_), altering calcium release and eventually leading to arrhythmias [26].

**Fig. 7:**
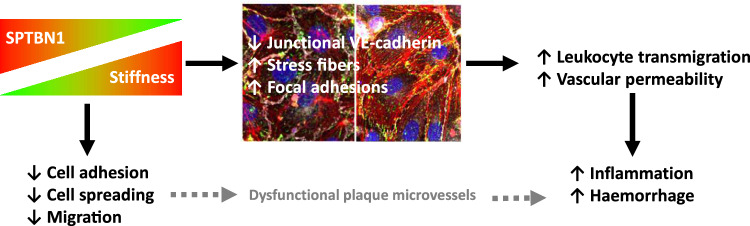
Schematic representation of the link between stiffness and SPTBN1 expression, and its effects in endothelial cells. SPTBN1 levels are reduced upon higher local tissue stiffness. Lower levels of SPTBN1 in turn lead to (1) reduced cell adhesion, spreading, and migration, which may lead to less developed plaque microvessels, and (2) altered endothelial junctions, more stress fibers, and more focal adhesions, which in turn enhance leukocyte transmigration and local vascular permeability. Together, this will lead to an increased local inflammatory response, as well as a higher occurrence of vascular leakage and intraplaque hemorrhage

Taken together, SPTBN1 exerts pleiotropic functions in membrane organization in several cell types. Recently, two reports already described a role of the spectrin cytoskeleton in leukocyte rolling [27] and mechanosensing in endothelial cells [28], in part via anchoring of CD44. In this study, we also show that SPTBN1 was central in our plaque microvessel associated gene network and, in vitro and in situ, exerts important regulatory functions in endothelial cell. In this regard, its activity reminisces of that of its family member SPTAN1, which has previously been described in regulating cell endothelial cell–cell contacts [29]. Based on our data, we propose that SPTBN1 silencing induces two major effects in endothelial cells (Fig. 7). First, knockdown cells show impaired adhesion and spreading capacity upon seeding. This was in conjunction with reduced migration capacity during wound healing and the hampered CVP development in zebrafish in vivo. SPTBN1 in endothelial cells therefore seems to be important for facilitating cell spreading and motion, as also was alluded to by GO term enrichment in the pathway analysis. Second, reduced expression of SPTBN1 increased permeability and leukocyte transmigration over an endothelial monolayer. This phenomenon could be linked to loss of VE-cadherin, the predominant cell junction molecule in vascular endothelial cells, at the cell–cell junction, and less linear endothelial junctions. In addition, focal adhesion numbers where significantly increased upon SPTBN1 knockdown in vitro. As we observed decreased mRNA and protein expression of SPTBN1 in ruptured versus stable advanced plaques and plaque microvessels, respectively, this suggests similar vessel destabilising effects in human disease. Despite reduced SPTBN1 levels, microvessel densities were increased in ruptured plaques. As SPTBN1 levels showed an inverse correlation with the extent of plaque hemorrhage, the predominant effect of lower SPTBN1 in human plaques is most likely microvessel hyperpermeability instead of sprouting.

Gene ontology studies show that SPTBN1 may be acting in (I) ER to Golgi vesicle-mediated transport [30], a notion that is underpinned by the high expression of SPTBN1 we observed in or near the ER and Golgi, and (II) in plasma membrane/cytoskeleton organization and actin binding [25, 26, 30, 31]. The latter suggest that SPTBN1 may be part of the adhesome [32, 33], which forms a crucial link between the plasma membrane and adaptor molecules on the one hand and the actin cytoskeleton on the other hand, and which includes molecules like VE-cadherin [34], or focal adhesion molecules like paxillin and vinculin [35]. The direct link to VE-cadherin that we found in our pull-down experiments was unexpected, even though SPTBN1 had previously been described in localization of E-cadherin to the lateral membrane in epithelial cells [23, 36]. Although the exact mechanism for the increase in focal adhesions remains unclear, it in part could explain the augmented leukocyte transmigration we observed [37]. This contractile endothelial phenotype may also explain the increase in stress fibres we observed upon knockdown of SPTBN1 [38], and is in conjunction with the more instable junctional phenotype.

The adhesome regulatory function of SPTBN1 may underlie the observed effects on vascular permeability in vitro* (*2.5-fold increase), and in patient tissue, i.e. the significant correlation between SPTBN1 levels and intraplaque hemorrhage in symptomatic lesions in three separate patient cohorts. Moreover, there was a strong enrichment of plasma proteins and hemorrhage GO terms among the MVD correlated peptides in the proteomics study. Moreover, we could attribute the reduction in SPTBN1 both in vitro, as well as in human plaque tissue, to increased tissue stiffness, the latter of which has been implicated in plaque destabilisation [39, 40]. Considering this strong correlation between SPTBN1 and a leaky vessel phenotype, the SPTBN1 axis may therefore be a potential important factor in determining the increased cardiovascular events by promoting hemorrhage, warranting further functional studies in vivo.

In conclusion, using a combination of histological analysis with genomic analyses we could show, that stiffness-dependent expression of SPTBN1 in atherosclerotic lesion microvessels may present a potential central factor involved in the leaky phenotype of these vessels. Intervening in this pathway may therefore be a way of selectively targeting the leaky vessel phenotype of plaque microvessels to prevent hemorrhage and further plaque exacerbation and adverse cardiovascular outcome.

### Supplementary Information

Below is the link to the electronic supplementary material.

Supplementary file1 (PDF 1023 kb)

Supplementary file2 (DOCX 65 kb)
