# Supplementary material for: Human atherosclerotic plaque transcriptomics reveals endothelial beta-2 spectrin as a potential regulator a leaky plaque microvasculature phenotype
Source: Angiogenesis. 2024 May 23;27(3):461–74. doi: 10.1007/s10456-024-09921-z (PMC11303431; doi:10.1007/s10456-024-09921-z)

Supplemental Table 1: qPCR primer sequences for *dock1*, *sptbn1* and *zeb1*

Gene	Forward/Reverse	Sequence
dock1	Forward	5’-gacagcaccccttccaga-3’
	Reverse	5’-ggacctggagtgcattttct-3’
sptbn1	Forward	5’-gagacagcggctcgagat-3’
	Reverse	5’-gtccataatgtagagcatttcctg-3’
zeb1	Forward	5’-atctcagtgttcttcaccgtctc-3’
	Reverse	5’-atctgtggtcgtgtgggact-3’

Supplemental Table 2: ranking of most central module L gene members, based on correlation to microvessel density (MVD) and centrality to the module.

Gene Symbol	EntrezID	Corr.MVD	p-value corr.MVD	Centrality	p-value centrality
abca9	10350	0.863	2.863E-08	0.839	1.610E-07
cask	8573	0.785	3.317E-06	0.836	1.910E-07
zeb1	**6935**	**0.781**	**4.129E-06**	**0.897**	**1.340E-09**
pfn2	5217	0.752	1.442E-05	0.822	4.644E-07
ppp2cb	5516	0.743	2.059E-05	0.886	3.754E-09
rpl32	6161	0.741	2.288E-05	0.911	2.447E-10
acsl3	2181	0.740	2.397E-05	0.867	2.056E-08
cav2	858	0.739	2.435E-05	0.938	4.541E-12
sptbn1	**6711**	**0.678**	**1.938E-04**	**0.830**	**2.796E-07**
dock1	**1793**	**0.572**	**2.840E-03**	**0.797**	**1.815E-06**

Supplemental Figure 1

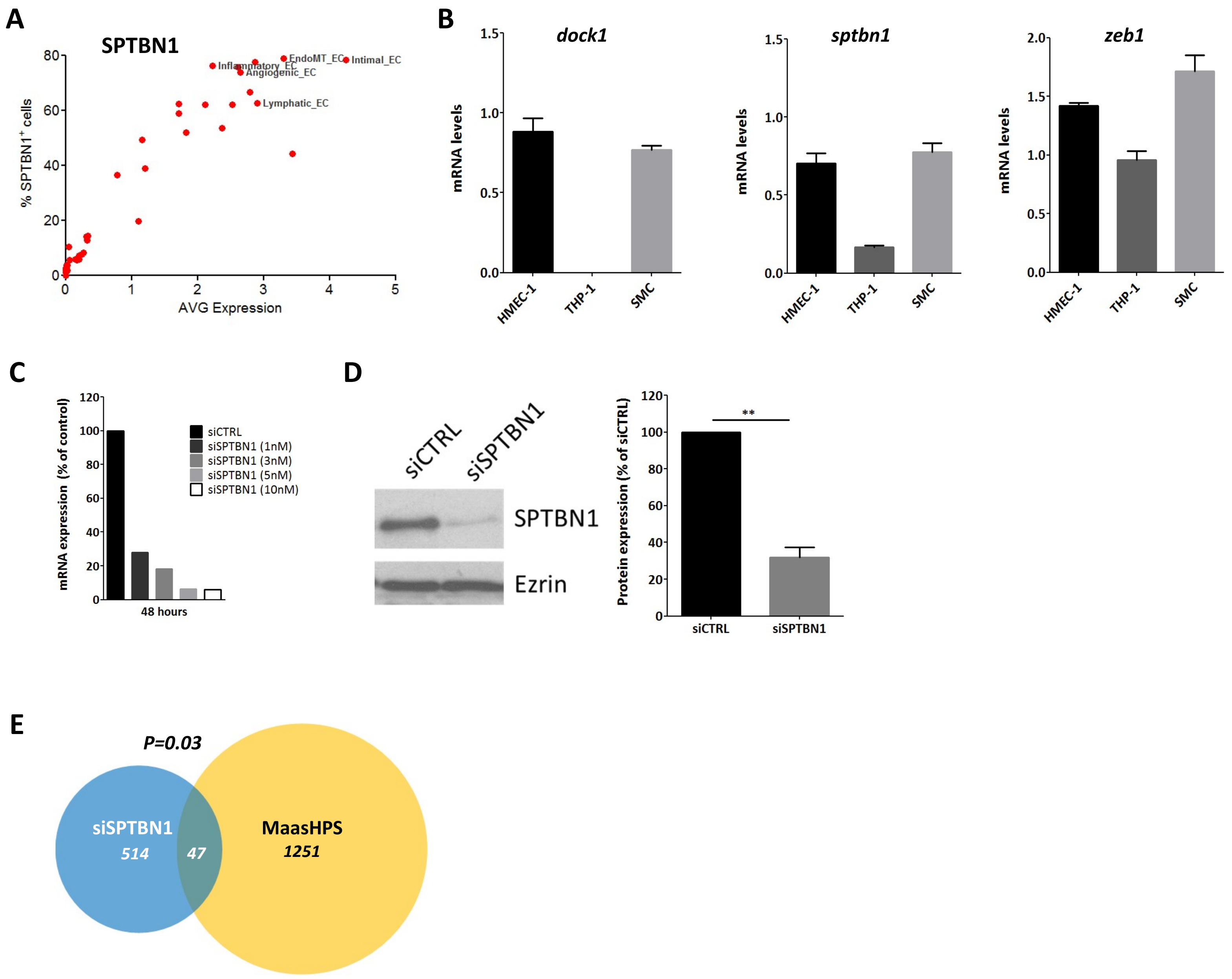

Supplemental Figure 2

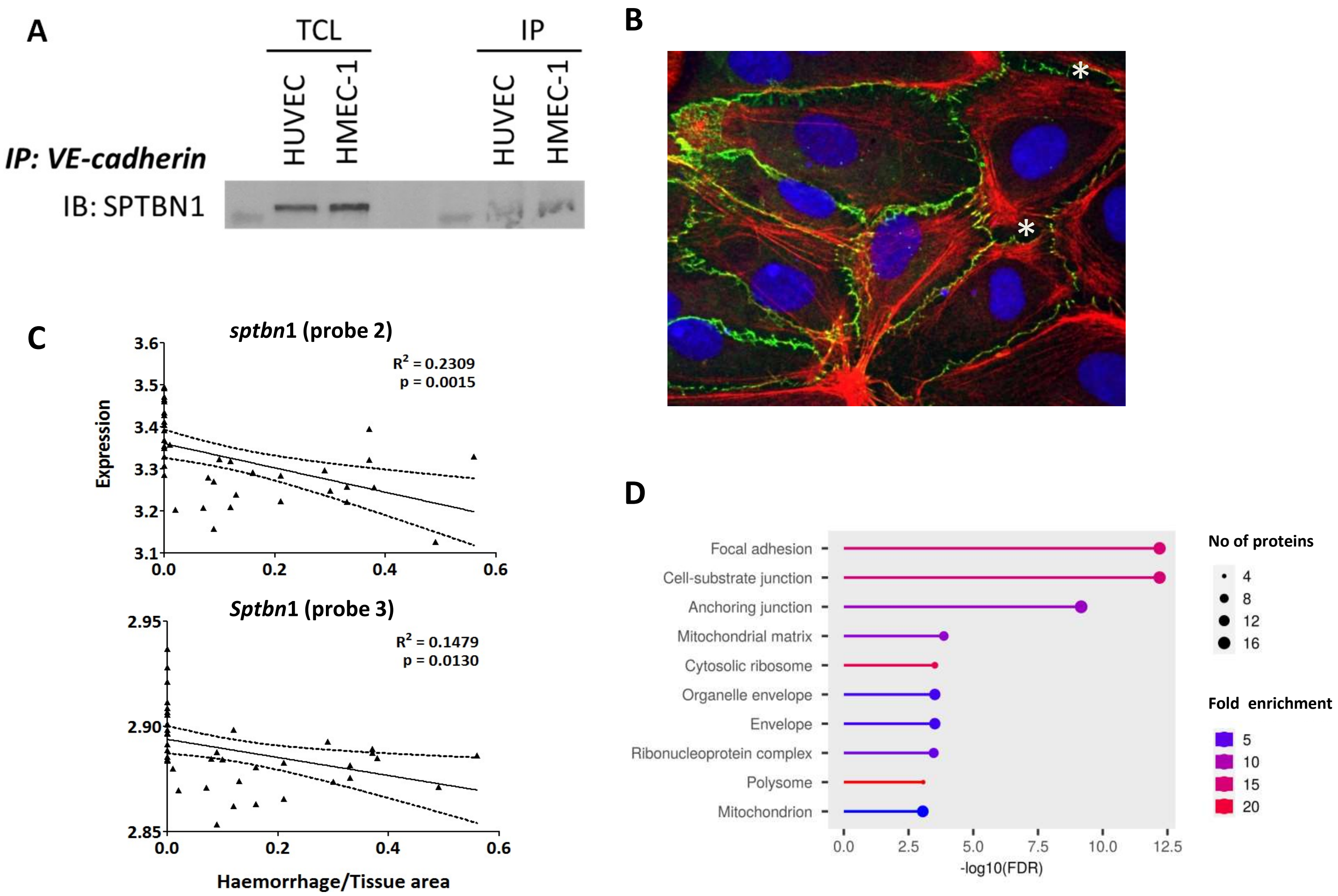

Supplement: Supplementary file 1 — Supplementary file1 (PDF 1023 kb) [file 10456_2024_9921_MOESM1_ESM.pdf]
