# Supplementary material for: Human atherosclerotic plaque transcriptomics reveals endothelial beta-2 spectrin as a potential regulator a leaky plaque microvasculature phenotype
Source: Angiogenesis. 2024 May 23;27(3):461–74. doi: 10.1007/s10456-024-09921-z (PMC11303431; doi:10.1007/s10456-024-09921-z)
Supplement: Supplementary file 2 — Supplementary file2 (DOCX 65 kb) [file 10456_2024_9921_MOESM2_ESM.docx]

**Extended materials & methods**

**Patient samples, histology and immunohistochemistry**

Human atherosclerotic plaque samples were obtained from carotid artery lesions from 24 patients undergoing endarterectomy (Department of Surgery, Zuyderland Medical Center, Sittard-Geleen, the Netherlands). The tissue was part of the Maastricht Pathology Tissue Collection (MPTC), and collection, storage, and use of tissue and patient data were performed in agreement with the Dutch Code for Proper Secondary Use of Human Tissue. This study complies with the Declaration of Helsinki, and the local Medical Ethical Committee in accordance with national regulations approved use of this tissue (protocol number 16-4-181). Immediately after resection, each atheroma was divided into parallel segments of 5 mm. Snap frozen segments for RNA isolation were alternated by formalin-fixed segments for histology and were only included when both adjacent haematoxylin-eosin (HE) stained sections were classified as advanced-stable or ruptured atherosclerotic lesions. Plaques were staged by histological analysis based on HE and Movat’s staining of adjacent slides according to Virmani *et al*, where pathological intimal thickening (PIT) was classified as early lesions, thick fibrous cap atheroma (TkFCA) were classified as advanced stable, and intraplaque hemorrhage (IPH) as ruptured (advanced unstable) segments, respectively (1). Two patients were excluded due to missing data. Patients were selected for having an advanced stable and ruptured snap- frozen segment within the lesion (advanced stable (n=21) and ruptured (n=23) segments).

Immunohistochemical stainings were performed on consecutive paraffin sections for vascular endothelial marker CD31 (Dako), the angiogenic marker CD105 (Thermo Scientific), the smooth muscle cell/pericyte αSMA (Dako), the macrophage marker CD68 (Dako), and SPTBN1 (Abcam). Appropriate IgG control antibodies were used as a negative control.

In all sections, the total amount of CD31^+^ microvessels, and CD68^+^ macrophages in the plaque was counted by two observers and corrected for plaque size, yielding the microvessel density (MVD; vessels/mm^2^), or lesional macrophage content (% of total plaque area). In addition, microvascular hotspots (>3 microvessels per highpower field (200x)), were counted (2).

To narrow down the target identification to intraplaque vessel function, not angiogenic regulators, we performed double staining for CD31/CD105 – to assess the percentage of angiogenic endothelium – and αSMA/CD31 (SMC coated microvessels; quantified morphometrically using Leica QWin V3.0 (Leica)). Furthermore double staining for CD31/SPTBN1 was performed. Double stainings were analyzed with the Nuance spectral imaging system (Caliper Life Science). Multispectral imaging (MSI) data sets were taken from 420‐720 nm at 20nm intervals using a DM‐5000 Leica microscope system at 20x (Plan Apo). Spectral libraries of single‐red (Vector Red), single‐blue (Vector Blue), single-brown (DAB), and natural occurring iron pigments were obtained from the control slides. The resulting spectral library was applied to spectrally unmix the double stained images into the individual components using the Nuance™ 3.0 software (3). Pseudo-color images showing co‐localization, as well as an exclusive image of co-localization, were generated with the Nuance 3.0 software. Quantitative assessment of co‐localization and pixel‐based measurement of the individual markers per microscopic field was done with the same software.

**Cell culture**

Human umbilical vein endothelial cells (HUVEC) or human microvascular endothelial cells (HMEC-1) were cultured on fibronectin (FN) coated plates in respectively EGM2 or RPMI1640+glutamax. HUVECs were used for experiments between P2 and P4, HMEC-1 cells were used until a maximum of P20. For stiffness experiments, cells were cultured on FN coated acrylamide stiffness gels of 2, 25, or 50kPa.

For knockdown of specific genes, cells were treated for 4 hours with a targeted siRNA in combination with HiPerfect transfection reagent in Optimem, and cells were used for various assays after 24-48 hours post-transfection. A scramble siRNA was used as a control, and knockdown was checked by Western Blot and qPCR.

For qPCR, RNA was isolated using the Trizol method, checked for concentration and purity, after which cDNA was synthesized, and analyzed using a BIO-RAD CFX96 Realtime System. Primer sequences are depicted in supplemental table 1. 18S RNA and Cyclophilin A were used as housekeeping genes.

**RNA extraction & transcriptomics on patient samples**

RNA isolation on whole plaque tissue sections was performed by Guanidium Thiocyanate lysis followed by Cesium Chloride gradient centrifugation. Following extraction, the RNA was further purified using the Nucleospin RNAII kit (Macherey-Nagel GmbH & Co. KG). RNA concentration was measured using a Nanodrop ND-1000 spectrophotometer (Nanodrop Technologies). The RNA quality and integrity was determined using Lab-on-Chip analysis on an Agilent 2100 Bioanalyzer (Agilent Technologies). The lowest RNA Integrity Number (RIN) was 5.6, the average RIN being 7.23 ± 0.48.

Biotinylated cRNA was prepared using the Illumina TotalPrep RNA Amplification Kit (Ambion, Inc.) according to the manufacturer’s specifications starting with 100 ng total RNA. 750 ng of cRNA per sample was used for hybridization. Hybridization to Illumina Human Sentrix-8 V2.0 BeadChip® and washing were performed according to the Illumina standard assay procedure. Scanning was performed on the Illumina BeadStation 500 (Illumina Inc.), image analysis and extraction of raw expression data was performed with Illumina Beadstudio v3 Gene Expression software with default settings (no background subtraction) and no normalization.

**Computational methods (patient samples)**

Analyses of transcriptomic data have been performed in R (4). Raw expression data were imported using the package lumi (5), and its implementation of Variance Stabilizing Transform was used for background correction. Data were normalized by robust spline normalization, and a filter was imposed to exclude all those probes whose detection call resulted ambiguous (30 or more readings of one probe with a p-value higher than 0.01).

Co-expression networks have been estimated by applying the methods implemented in the package WGCNA (6), setting Softhreshold Power at 10. Modules, clusters of densely interconnected genes, were identified using the default method of hierarchical clustering (7) and the function ‘cutreeDynamic’(8), with ‘minModuleSize=30’ and ‘deepSplit=2’, to "cut" the branches of the tree. The function ‘moduleEigengenes’ was used to calculate modules eigenvalues, as estimates of central behaviour, and the function ‘mergeCloseModules’ to optimize the heuristics of modules by merging those who’s eigenvalues were very highly correlated, to make their interpretation more straightforward.

Exploiting the availability of plaques classified as advanced stable, and ruptures, as described above, we searched for consensus modules, those whose overall composition was preserved across the two conditions, by the function ‘blockwiseConsensusModule’. We assumed that consensus gene clusters would be reflective of biological processes underlying plaque progression, whilst modules only identified in ruptured samples would be reflective of the changes happening during rupture and the consequent repair (hence metastability clusters).

Module-traits association was estimated by Pearson correlation of the expression modules eigenvalues and quantitative traits, among which quantitative estimates of CD31^+^, CD105^+^CD31^+^, and αSMA^+^.CD31^+^ from histological staining of the plaques were of primary interest to us, setting a cut-off at p-value≤0.05. Within each module related to traits, genes with high module membership were considered for further investigation, as suggested by Langfelder and Horvath (9, 10). Hereafter, we exported the network data to Cytoscape for visualization and further analyses (11). The plugin ClueGO was used to enrich the modules of interest for over-represented Gene Ontology terms and Pathways(12).

In order to reveal the relationships between proteins/peptides and MVD, a ranking list was performed to show the importance of proteins/peptides relating to the MVD based on two different measurements, the Pearson Correlation Coefficient and the Maximal Information Coefficient (MIC) (13). In each ranking list, top-100 high-ranked proteins/peptides were listed, after which overlapping proteins/peptides were marked and cross-referenced with haemorrhage related proteins.

**RNA extraction & transcriptomics on *in vitro* samples**

RNA isolation was performed by Guanidium Thiocyanate lysis followed by Cesium Chloride gradient centrifugation (n=3 independent experiments, n=3 per experiment). Following extraction, the RNA was further purified using the Nucleospin RNAII kit (Macherey-Nagel GmbH & Co. KG). RNA concentration was measured using a Nanodrop ND-1000 spectrophotometer (Nanodrop Technologies). The RNA quality and integrity was determined using Lab-on-Chip analysis on an Agilent 2100 Bioanalyzer (Agilent Technologies). The lowest RNA Integrity Number (RIN) was 5.1, the average RIN being 6.98 ± 1.53.

Biotinylated cRNA was prepared using the TargetAmp-Nano Labeling Kit for Illumina Expression BeadChip (epicenter, Illumina) according to the manufacturer’s specifications starting with 500 ng total RNA. 750 ng of cRNA per sample was used for hybridization. Hybridization to Illumina HumanHT-12 v4.0 BeadChip® and washing were performed according to the Illumina standard assay procedure. Scanning was performed on the Illumina BeadStation 500 (Illumina Inc.), image analysis and extraction of raw expression data was performed with Illumina GenomeStudio V2011.1 software with default settings (no background subtraction) and no normalization.

Probe-level expression data for all samples (3-4 replicates per group) was processed and log2-transformed using the bead array software package available via the R/Bioconductor programming interface (14, 15). All samples passed quality control based on internal control probes as recommended by the manufacturer and quality metrics implemented in the arrayQualityMetrics package (16).. Expression data was quantile-normalized and analyzed for differential expression between sample groups using the limma package (17). Genes were considered as differentially expressed if they show expression fold-changes > 1.2 or < 0.83 and FDR-adjusted p-values < 0.05. Differentially expressed genes were analyzed for enrichment of KEGG pathways and gene ontology (GO) terms using either the top 100 differentially expressed genes for overrepresentation analysis or the complete gene list ordered by p-value for gene set enrichment analysis as implemented in the clusterProfiler package (18).

**Immunofluorescence & whole mount staining**

Immunofluorescent staining was performed on HUVEC or HMEC-1 cells. Cells were fixed in 3.7% paraformaldehyde (PFA) for 10 minutes, permeabilized, and blocked using 5%BSA in PBS+ (PBS supplemented with 0.5mM MgCl_2_ and 1mM CaCl_2_). Primary antibody incubation (VE-cadherin, SPTBN1, ZO-1, p-paxillin) was performed for 1 hour. After washing, secondary antibody and phalloidin staining, for staining F-actin, was performed for 45 minutes. Finally, cells were stained with Hoechst or DRAQ5 for nuclear staining and mounted in Mowiol. Appropriate IgG control antibodies were used as a negative control.

Whole mount staining on human vessels segments was performed as described by Van Geemen et al (19). Briefly, mammary vessels (artery and vein) were isolated from remaining tissue of patients (n=4) that underwent breast reconstruction in the Antoni van Leeuwenhoek Hospital (Amsterdam, the Netherlands). All vessels were obtained with informed consent and according the (Dutch) guidelines for secondary used materials. Vessels were fixed in 3.7% PFA for 10 minutes within 24 hours after collection. After preparation, vessels were permeabilized and stained as described above. All imaging was performed on a Leica SP8 confocal system using a 20x or 40x objective. Focal adhesion data (number of focal adhesion, focal adhesion size) was analysed using ImageJ.

**Functional *in vitro* assays**

For measuring EC adhesion (n=3 independent experiments, n=6 per experiment), HMEC-1 cells (2.5x10^4^ cells) were plated on a 96-well plate, and left to adhere for up to 4 hours. After adhesion period, non-adhered cells were washed away in PBS, and adherent cells were fixed in 1%PFA. Samples were stained with 0.1% crystal violet/20% methanol for 30 min, after which 10% acetic acid was added, and the extinction was measured at 595nm.

Cell spreading was assessed by subconfluent culture of HMEC-1 cells and subsequent staining of α-actinin (n=3 independent experiments, n=3 per experiment). Images were taken at 10x magnification, after which cell surface area was analyzed using Leica Qwin 3.1.

Wound healing assay was used to study EC migration (n=3 independent experiments, n=10 per experiment). Briefly, HMEC-1 cells (1.0x10^4^ cells) were seeded on a coated 96-well plate. Upon confluency, a scratch was applied, and cell migration was measured for 16 hours on a Zeiss Observer Z1 microscope using a 10x objective. All live imaging was performed at 37 °C in the presence of 5% CO_2_. Time until closure of the wound, as well as migration speed of the ECs, was determined using ImageJ.

To assess EC proliferation, ECs (5.0x10^3^ cells) were seeded in a 96-well plate (n=3 independent experiments, n=10 per experiment). Upon 70% confluency, cells were cultured with [^3^H]-thymidine for 6 hours, after which medium was removed and plate was stored at -20°C. Proliferation was then determined using liquid scintillation counting. For cell cycle analysis, HMEC-1 cells were grown up to 70% confluency, after which medium was removed, 70% ethanol was added, and cells were incubated at -20°C for 2 hours (n=3 independent experiments, n= 8 per experiment). Cells were permeabilized and stained with Propidium Iodide (PI) after which cell cycle was determined by flow cytometry.

To determine possible effects on the blood vessel formation, tube formation capacity was assessed. HMEC-1 cells (2.5x10^4^ cells) were cultured in a 96 well plate on Matrigel (BD Matrigel Matrix) for up to 24 hours (n=3 independent experiments, n= 6 per experiment). Cells were fixed using 1% PFA, pictures were taken at 10x magnification, and variables of tube formation (tube length, number of tubes, number of loops, number of branch points, and number of branches/branch point) were analyzed using Leica Qwin 3.1.

Vascular permeability was assessed using a Transwell assay and ECIS. For Transwell assay, HUVEC and HMEC-1 cells were cultured on a FN-coated Transwell filter (3µm pore size) and grown until confluency. To assess permeability, 70kD and 150 kD dextran (TxRed and FITC, respectively) were added to the top compartment, and fluorescence increase in the lower compartment was measured live for up to 60 minutes (n=4 independent experiments, n=4 per experiment). Alternatively, permeability was assessed by measuring electrical resistance using ECIS as described previously (20) (n=3 independent experiments, n=2 per experiment). Electrode-arrays (8W10E; IBIDI, were treated with 10 mM l-cysteine (Sigma) for 15 min at 37°C and subsequently coated with 10 μg/ml fibronectin (Sigma) in 0.9% NaCl for 1 h at 37°C. Cells were seeded at 100,000 cells per well (0.8 cm^2^) and grown to confluence. Electrical resistance was continuously measured at 37°C under 5% CO_2_ using ECIS Z (Applied BioPhysics).

Neutrophil transmigration under flow was studied as described previously (21) (n=3 independent experiments, n= 3 per experiment). Briefly, HUVECs were cultured and transfected in a FN-coated ibidi μ-slide VI^0.4^ (iBidi) and stimulated overnight with TNFα (10 ng/ml) the day prior to the experiment. Neutrophils were freshly isolated from whole blood from healthy donors using a Percoll separation gradient. HUVECs were then exposed to 0.5 ml min^−1^ shear flow for 10 min (0.8 dyne per cm^2^) after which 2 × 10^6^ neutrophils were injected into the perfusion system and real-time leukocyte–endothelial interactions were recorded for 20 min by a Zeiss Observer Z1 microscope using a 20x objective, at 37 °C in the presence of 5% CO_2_. Transmigrated neutrophils were distinguished from those adhering to the apical surface of the endothelium by their transition from bright to phase-dark morphology. Percentage adherent or transmigrated neutrophils were manually quantified using the ImageJ plug-in Cell Counter.

**Immunoprecipitation and Western Blot**

For immunoprecipitation (IP), cells were washed twice with ice-cold PBS, supplemented with 1 mM CaCl_2_ and 0.5 mM MgCl_2_, and lysed in cold NP-40 lysis buffer (25 mM Tris, 100 mM NaCl, 10 mM MgCl_2_, 10% (v/v) glycerol and 1% (v/v) Nonidet P-40, pH 7.4) supplemented with a phosphatase inhibitor cocktail (Sigma) and fresh protease-inhibitor-mixture tablets (Roche Applied Science). After 10 min, cell lysates were collected and centrifuged at 14,000 g for 10 min at 4°C. The supernatant was incubated with 0.5 μg VE-cadherin antibody (clone BV6, Millipore) or 1 μg SPTBN1 antibody (Abcam) and 30 µl of protein-G-Dynabeads at 4°C with continuous mixing for 2 hours. Subsequently, beads were separated using a magnet at 4°C, washed five times with NP-40 lysis buffer and boiled in SDS sample buffer containing 4% β-mercaptoethanol.

Samples were analyzed by using SDS-PAGE, 7.5% or 10% gels. Proteins were transferred onto a 0.2 µm nitrocellulose membrane (Whatman, Dassel, Germany), subsequently blocked with 5% (w/v) milk powder in Tris-buffered saline with Tween20 (TBST). The nitrocellulose membrane was incubated with specific primary antibodies (SPTBN1, VE-cadherin, ZO-1, paxillin) overnight at 4°C, followed by incubation with secondary HRP-labelled antibodies for 1 hour at room temperature. Actin or Ezrin were used as loading controls as indicated. Between the incubation steps, blots were washed with TBST. Staining was visualized with an enhanced chemiluminescence (ECL) detection system (ThermoScientific, Amsterdam, The Netherlands) for IP experiments, or normal ECL (ThermoScientific, Amsterdam, The Netherlands) for all other experiments. Analysis was performed using ImageJ.

For the patient samples, plaque protein lysates of 20 vs. 20 individuals retrieved from the Athero-Express study(22), which did or did not get a second cardiac event, were used for analysis of SPTBN1 protein expression using SDS-PAGE. In total 25 µg of plaque protein lysate was loaded (4-20% precast gel, Expressplus M42012) and SPTBN1 protein was detected using the primary antibody rabbit anti-human SPTBN1 (Abcam ab124888, 1:100). Visualization was achieved using West Femto Maximum Sensitivity ECL (Thermo Fisher, 34095) and protein expression was analyzed using ImageJ software corrected for PonceauS loading and normalized to the same control sample present on each blot.

**BiKE cohort**

The second cohort used for validation was the BiKE cohort. Patients undergoing surgery for high-grade (>50% NASCET)^(23)^ carotid stenosis at the Department of Vascular Surgery, Karolinska University Hospital, Stockholm, Sweden were consecutively enrolled in the study and clinical data recorded on admission. Symptoms (S) were defined as transitory ischemic attack (TIA), minor stroke (MS) and *amaurosis fugax* (retinal TIA). Patients without qualifying symptoms within 6 months prior to surgery were categorised as asymptomatic (AS) and indication for CEA based on results from the Asymptomatic Carotid Surgery Trial (ACST)^(24)^. Carotid endarterectomies (carotid plaques, CP) were collected at surgery and retained within the **Bi**obank of **K**arolinska **E**ndarterectomies (BiKE). The BiKE study cohort demographics, details of sample collection, processing and large-scale analyses (transcriptomic and proteomic profiling) were as previously described in details^(25-27)^. The microarray dataset is available from Gene Expression Omnibus (accession nr GSE21545). Briefly, for microarrays n=127 plaques were divided transversally at the most stenotic part, the proximal half of the lesion used for RNA preparation while the distal half was processed for histology. For LC-MS/MS proteomic analyses, atherosclerotic plaques from n=18 patients (matched for male gender, age and statin medication) were collected and processed as described earlier^(27)^. A central portion of the plaque corresponding to the maximum stenosis was separated from the respective downstream peripheral end (adjacent tissue) and used in comparisons. Normal artery controls (NA) were nine macroscopically disease-free iliac arteries and one aorta, obtained from organ donors without history of cardiovascular disease. All samples were collected with informed consent from patients or organ donors’ guardians. All human studies were approved by the regional Ethical Committee.

**Statistical and bioinformatic analyses BiKE**

Transcriptomic and proteomic dataset analyses were performed with GraphPad Prism 6.0 and Bioconductor using a linear regression model adjusted for age and gender or a two-sided Student’s t-test assuming non-equal deviation, with correction for multiple comparisons according to Bonferroni, as previously described(25, 27). Gene set enrichment analyses on Gene Ontology (GO) terms were performed using ENRICHR (<http://amp.pharm.mssm.edu/Enrichr>) software. Pearson correlations were calculated to determine the association of protein levels from LC-MS/MS data using Morpheus (<http://software.broadinstitute.org/morpheus>). In all analyses p<0.05 was considered to indicate statistical significance.

**Zebrafish**

*Tg(fli1a:eGFP)y1* embryos were injected with control morpholino (Ctrl Mo) or with morpholino against SPTBN1 (7.5ng/microliter) at the one-cell stage (N=35 per group). Confocal pictures of the caudal vasculature was taken at 48 hpf using a Zeiss confocal microscope. CVP thickness and area were analyzed using ImageJ software.

**Statistical analysis**

Unless explicitly specified otherwise, all data are presented as mean ± SEM. For patient data, groups were compared using a Mann-Whitney rank-sum test for continuous variables. For correlation analyses of human qPCR and protein expression data, Shapiro-Wilk test for normality was performed, after which correlations of Gaussian distributed data were calculated by Pearson and of non-Gaussian data by Spearman correlation test. For group comparisons, data were tested for Gaussian distribution, after which a Student t-test (Gaussian) or Mann-Whitney U test (non-Gaussian) was used to compare individual groups; multiple groups were compared by ANOVA or Kruskall-Wallis tests, with Bonferroni or Dunn’s post-hoc test, respectively. Statistics were performed using Graphpad Prism 7.0. A p-value of <0.05 was considered statistically significant. *denotes p≤0.05, **denotes p≤0.01, ***denotes p≤0.005, ****denotes p≤0.001.

**Extended references**

1. Virmani R, Kolodgie FD, Burke AP, Farb A, Schwartz SM. Lessons from sudden coronary death: a comprehensive morphological classification scheme for atherosclerotic lesions. Arterioscler Thromb Vasc Biol. 2000;20(5):1262-75.

2. Sluimer JC, Kolodgie FD, Bijnens AP, Maxfield K, Pacheco E, Kutys B, et al. Thin-walled microvessels in human coronary atherosclerotic plaques show incomplete endothelial junctions relevance of compromised structural integrity for intraplaque microvascular leakage. J Am Coll Cardiol. 2009;53(17):1517-27.

3. van der Loos CM. Multiple immunoenzyme staining: methods and visualizations for the observation with spectral imaging. J Histochem Cytochem. 2008;56(4):313-28.

4. Team RdC. R: A language and environment for statistical computing. In: R foundation for Statistical Computing V, Austria, editor. 2010.

5. Mason C, Dunnill P. A brief definition of regenerative medicine. Regenerative Medicine. 2008;3(1):1-5.

6. Langfelder P, Horvath S. WGCNA: an R package for weighted correlation network analysis. BMC Bioinformatics. 2008;9:559.

7. Ray SS, Bandyopadhyay S, Pal SK. Gene ordering in partitive clustering using microarray expressions. J Biosci. 2007;32(5):1019-25.

8. Langfelder P, Zhang B, Horvath S. Defining clusters from a hierarchical cluster tree: the Dynamic Tree Cut package for R. Bioinformatics. 2008;24(5):719-20.

9. Langfelder P, Horvath S. Eigengene networks for studying the relationships between co-expression modules. BMC Syst Biol. 2007;1:54.

10. Langfelder P, Luo R, Oldham MC, Horvath S. Is my network module preserved and reproducible? PLoS Comput Biol. 2011;7(1):e1001057.

11. Shannon P, Markiel A, Ozier O, Baliga NS, Wang JT, Ramage D, et al. Cytoscape: a software environment for integrated models of biomolecular interaction networks. Genome Res. 2003;13(11):2498-504.

12. Bindea G, Mlecnik B, Hackl H, Charoentong P, Tosolini M, Kirilovsky A, et al. ClueGO: a Cytoscape plug-in to decipher functionally grouped gene ontology and pathway annotation networks. Bioinformatics. 2009;25(8):1091-3.

13. Reshef DN, Reshef YA, Finucane HK, Grossman SR, McVean G, Turnbaugh PJ, et al. Detecting novel associations in large data sets. Science. 2011;334(6062):1518-24.

14. Dunning MJ, Smith ML, Ritchie ME, Tavare S. beadarray: R classes and methods for Illumina bead-based data. Bioinformatics. 2007;23(16):2183-4.

15. Gentleman RC, Carey VJ, Bates DM, Bolstad B, Dettling M, Dudoit S, et al. Bioconductor: open software development for computational biology and bioinformatics. Genome Biol. 2004;5(10):R80.

16. Kauffmann A, Gentleman R, Huber W. arrayQualityMetrics--a bioconductor package for quality assessment of microarray data. Bioinformatics. 2009;25(3):415-6.

17. Ritchie ME, Phipson B, Wu D, Hu Y, Law CW, Shi W, et al. limma powers differential expression analyses for RNA-sequencing and microarray studies. Nucleic Acids Res. 2015;43(7):e47.

18. Yu G, Wang LG, Han Y, He QY. clusterProfiler: an R package for comparing biological themes among gene clusters. OMICS. 2012;16(5):284-7.

19. van Geemen D, Smeets MW, van Stalborch AM, Woerdeman LA, Daemen MJ, Hordijk PL, et al. F-actin-anchored focal adhesions distinguish endothelial phenotypes of human arteries and veins. Arterioscler Thromb Vasc Biol. 2014;34(9):2059-67.

20. Timmerman I, Heemskerk N, Kroon J, Schaefer A, van Rijssel J, Hoogenboezem M, et al. A local VE-cadherin and Trio-based signaling complex stabilizes endothelial junctions through Rac1. J Cell Sci. 2015;128(18):3514.

21. Kroon J, Daniel AE, Hoogenboezem M, van Buul JD. Real-time imaging of endothelial cell-cell junctions during neutrophil transmigration under physiological flow. J Vis Exp. 2014(90):e51766.

22. Hellings WE, Moll FL, de Kleijn DP, Pasterkamp G. 10-years experience with the Athero-Express study. Cardiovasc Diagn Ther. 2012;2(1):63-73.

23. Naylor AR, Rothwell PM, Bell PR. Overview of the principal results and secondary analyses from the European and North American randomised trials of endarterectomy for symptomatic carotid stenosis. Eur J Vasc Endovasc Surg. 2003;26(2):115-29.

24. Halliday A, Harrison M, Hayter E, Kong X, Mansfield A, Marro J, et al. 10-year stroke prevention after successful carotid endarterectomy for asymptomatic stenosis (ACST-1): a multicentre randomised trial. Lancet. 2010;376(9746):1074-84.

25. Perisic L, Aldi S, Sun Y, Folkersen L, Razuvaev A, Roy J, et al. Gene expression signatures, pathways and networks in carotid atherosclerosis. J Intern Med. 2016;279(3):293-308.

26. Perisic L, Hedin E, Razuvaev A, Lengquist M, Osterholm C, Folkersen L, et al. Profiling of atherosclerotic lesions by gene and tissue microarrays reveals PCSK6 as a novel protease in unstable carotid atherosclerosis. Arterioscler Thromb Vasc Biol. 2013;33(10):2432-43.

27. Perisic Matic L, Rykaczewska U, Razuvaev A, Sabater-Lleal M, Lengquist M, Miller CL, et al. Phenotypic Modulation of Smooth Muscle Cells in Atherosclerosis Is Associated With Downregulation of LMOD1, SYNPO2, PDLIM7, PLN, and SYNM. Arterioscler Thromb Vasc Biol. 2016;36(9):1947-61.
